# Gender differences in sex life issues – A population-based study of migraine sufferers

**DOI:** 10.1186/1471-2296-9-19

**Published:** 2008-04-09

**Authors:** Markku PT Sumanen, Ansa Ojanlatva, Anna Rantala, Lauri H Sillanmäki, Kari J Mattila

**Affiliations:** 1 University of Tampere, Medical School, Finland; 2 Kangasala Health Centre, Finland; 3 Department of Teacher Education, Turku, Finland; 4 Institute of Biomedicine, Center for Reproductive and Developmental Medicine, University of Turku, Finland; 5 Turku City Hospital, Finland; 6 Tampere Health Centre, Finland; 7 University of Turku, Department of Public Health, Finland; 8 Hospital District of Pirkanmaa, Department of General Practice, Finland

## Abstract

**Background:**

Migraine is considered to have a negative influence on sex life. The present study was to analyse the perceptions of importance of and satisfaction with sex life as well as the expression of interest in sex among people having migraines in a prospective follow-up mail survey in 1998 and 2003.

**Methods:**

The random sample was stratified according to gender and age in four age groups (20–24, 30–34, 40–44, and 50–54 years). Altogether 25 898 individuals responded to the baseline and 19 626 to the follow-up questionnaire (75.8% response rate). We examined as to how the perceptions of sex life of those suffering from migraine changed during a 5-year follow-up. Conditional logistic regression was used to analyse the data of the responses on self-reported migraine in the baseline and follow-up surveys (N = 2 977, 79.2% women). Each person with migraine was assigned a gender- and age-matched control in the analysis.

**Results:**

All three outcome variables tended to decrease in value. Importance of sex life was higher among men with migraine than among their controls. Among women migraine lessened interest in sex life.

**Conclusion:**

Our findings suggested that migraine has a different impact on sex life among women from that among men.

## Background

Headache has wide-ranging adverse effects and is often accompanied by considerable worry [1]. The majority of migraine sufferers have acknowledged that migraine has a significant effect on both activity-based quality of life and on personal relationships [2]. Most of the migraine sufferers are able to cite negative or unconstructive family situations [3] and/or interference with spousal relationships as a result of their migraines. Moreover, social factors during childhood are likely to be associated with migraine headaches during adulthood [4]. In addition to headache contributing to less sexual activity or sexual activity causing headaches [5], some headache sufferers have reported marked sexual arousal during a migraine attack [6]. Being involved in or having sex may in fact provide some relief for migraine sufferers [7]. Migraine sufferers may have elevated levels of sexual desire and they may be the least likely ones to elude sexual activity [8].

The diagnosis of a migraine headache is a reason for other medical concerns [9]. Lifetime prevalence of major depression has been noted approximately three times more often among migraine sufferers than among their controls [10]. The association of serotonin and depression is well-known and connections exist between serotonin and migraine attacks [11-14]. Serotonin is also involved in libido. Already decades ago, an absolute or relative excess of serotonin in the midbrain was reported to antagonize testosterone which in turn causes decreased libido among men [15]. The association between depression and libido has been confirmed [16-18]. Among women, the role of testosterone is perceived to be less obvious while migraines have been found to be associated with hormones. Female hormones have been suggested to have an important factor responsible for the gender differences in headache disorders [19-22]. The associations between migraines, serotonin metabolism and sex life may be explained, but does migraine affect sex life?

The present study was to analyse the perceptions of importance of and satisfaction with sex life as well as the expression of interest in sex among people having migraines in a prospective follow-up mail survey.

## Methods

Health and Social Support Study (the HeSSup Study) is a prospective mail survey designed to follow up a large number of diverse issues within the Finnish working-aged population. The random sample (N = 64 797) was obtained from the Finnish Population Register Centre and stratified according to gender and four age groups (20–24, 30–34, 40–44, and 50–54 years). A total of 25 898 individuals (40% response, 59% women) returned the baseline survey questionnaire in 1998. Data were found to be representative of the Finnish working-aged people [23]. All those who responded to the first questionnaire received the follow-up questionnaire in 2003 and this survey yielded 19 629 respondents (75.8% response rate, 61.4% women). During the 5-year follow-up period 1.9% of the random sample and 1.3% of individuals responding to the baseline survey had died.

The HeSSup study used survey data with healthy subjects. Consequently, the medical ethics committee of the Turku University Central Hospital certified that no committee approval was necessary according to the present Finnish law. The responding individuals gave their informed consent with signature so that personal information may be linked via registries. The voluntary response was adequate.

### Questionnaire

The participants were asked whether 'they presently had or had ever had migraine' as verified by a physician. This diagnosis was based on the personal perception of whether they did or did not have migraines and not obtained from the medical case history. In effort to verify the method of diagnosing self-reported migraine, we compared the prevalence of migraine among the health care staff of nurses and physicians (n = 688) and other people in the baseline data. The prevalence rates were 18.6% for the health care staff and 19.1% for the total HeSSup baseline population. The participants were also inquired as to how important sex life was to them and whether they were or were not satisfied with their sex life. These personal perceptions were examined with a Likert-type 7-point scale, but only 3 categories were used (1–2, 3–5, and 6–7 combined) [24,25].

The questionnaire contained an item from the Beck Depression Inventory (BDI) [26]. The single question measured interest in sex with four response alternatives (1 = no recent change in interest in sex, 2 = less interested, 3 = much less interested, 4 = lost interest completely). We considered it acceptable to make use of this item; it has been used in two previous studies [24,25]. The responses were grouped into two new categories (2–4 combined) to establish whether interest in sex did or did not decrease.

We analysed the responses of those who affirmatively replied to the question on self-reported migraine in both the baseline and follow-up questionnaires. Altogether 2 977 respondents indicated migraine in both questionnaires (79.2% women). One age- and gender-matched control was selected for every individual with migraine from among those who reported having migraine in neither questionnaire.

### Statistical analyses

Conditional regression analysis was used to treat the data. The results were expressed as odds ratios with 95% confidence intervals. Associations with p-values <0.05 were interpreted as statistically significant. In the perceptions of importance of and satisfaction with sex life, the response options of 'fairly' and 'not important/satisfied' were combined and they were compared with the response options of 'very important/satisfied'. The statistical analyses were performed using the SAS System for Windows (release 9.1.3.).

## Results

All three outcome variables tended to decrease in value in the present 5-year follow-up study. Importance of sex life decreased among both men and women having migraine but even more so among women (Table 1). The proportions of those reporting satisfaction with sex life decreased as well. The response categories of 'very important' and 'very satisfied" declined in frequency. The greatest change from 1998 to 2003 was found in interest in sex life (8.5%).

**Table 1:** Proportions (%) of answers to questions on sex life issues to migraine patients in the years 1998 and 2003.

	Women (N = 2358)			Men (N = 619)		
		
	1998	2003	change	1998	2003	Change
Importance of sex life						
not important	7.9	12.5	4.6	4.7	6.0	1.3
fairly important	42.3	44.8	2.5	25.6	28.1	2.5
very important	49.9	42.7	-7.2	69.7	65.9	-3.8
Satisfaction with sex life						
not satisfied	11.8	15.6	3.8	15.2	15.7	0.5
fairly satisfied	39.3	40.6	1.3	38.0	43.9	5.9
very satisfied	48.9	43.8	-5.1	46.8	40.5	-6.3
Interest in sex life						
No recent change	62.1	53.8	-8.3	74.8	66.3	-8.5

In the conditional regression analysis, the associations of importance of sex life were inclined to be higher among men with migraine than among their controls for the baseline and follow-up questionnaires, whereas no such associations were evident among women with migraine (Table 2). No associations of satisfaction with sex life were observed for the two questionnaires among men or women. Migraine was associated with lessening interest in sex life among women in both the baseline and follow-up data but not among men.

**Table 2:** Age- and gender-matched ORs with 95% CI in conditional logistic analysis in the years 1998 and 2003 to questions on sex life issues to migraine patients and controls. Statistical significant results in bold.

	Women		Men	
		
	Year 1998	Year 2003	Year 1998	Year 2003
Importance of sex life				
Not or fairly important vs very important	0.94 (0.84 – 1.06)	1.03 (0.92 – 1.16)	**0.70 (0.55 – 0.89)**	**0.78 (0.62 – 0.98)**
Satisfaction with sex life				
Not or fairly satisfied vs very satisfied	0.98 (0.87 – 1.10)	1.03 (0.92 – 1.16)	0.88 (0.70 – 1.10)	0. 91 (0.73 – 1.14)
Interest in sex life				
No recent change vs decreased interest	**0.84 (0.74 – 0.95)**	**0.83 (0.74 – 0.94)**	0.85 (0.65 – 1.12)	0.82 (0.64 – 1.05)

## Discussion

The present study was to analyse the perceptions of importance of and satisfaction with sex life as well as the expression of interest in sex among men and women suffering from migraine in a prospective follow-up mail survey. There was decreasing interest in sex life among women and increasing importance of sex life among men.

The method of assessment was based on a question that inquired the personal perception of having or not having migraine, a type of headache, in the mail survey. Frequency or intensity of the migraine attacks was not known. We did not study the other forms of headache. Responses were analysed only when the question was responded to in both the baseline and follow-up questionnaires. One person in four having migraine did not respond to the follow-up survey but the response rate may be considered high. The non-response in the follow-up data did not have a significant effect on the outcomes and the data may be considered reliable.

Being a question of self-report, it is not absolutely certain whether migraine did or did not exist as a form of headache. There is no specific test to verify the diagnosis and many people having migraines do not consult a physician [27], which is to underline as to how difficult it is to establish a reliable migraine diagnosis. We venture to state that people having migraines really do suffer from this type of headache and that the mail survey may be considered a valid method of data gathering since the medical staff of nurses and physicians also responded in the same manner as the rest of the participants did.

In a recent study migraine did not affect sex life [8]. In the present study, no significant association of satisfaction with sex life was observed. Sexual satisfaction may be evident when there is a balance between what one contributes to a relationship and receives from it in return. In so doing, it may be assumed that there was equity, fair play in these relationships. On the other hand, migraine was a contributor to increasing importance of sex life among men and lessening interest in sex among women. The traditional way is to look at the biological processes and to explain sexuality issue with them while there are efforts underway to discover how behaviour and experience in turn affect the biological processes. Women's sexual behaviours are more complicated than men's sexual behaviours and influenced by relationship issues.

Why then does interest in sex life surface as an issue of lessening interest in sex among women and why does importance of sex life increase among men alone? Age is being indicated as a potential contributor in the prevalence of at least some of the known sexual problems. Lubrication is known to reduce in quantity with age among women and makes a difference in interest in sex. The Global Study of Sexual Attitudes and Behaviours (GSSAB) on sex and relationships of men and women for 40–80 years of age (13 882 women and 13 618 men from 29 countries) indicated that age is an important correlate of lubrication difficulties among women and of several sexual problems, including a lack of interest in sex or the inability to reach orgasm. Erectile difficulties were indicated among men. The study concluded that sexual problems tend to be more associated with physical health and aging among men than women [28]. It is also possible that women feel more ill and suffer from a migraine attack more than men do. This may be associated with a decreasing interest in sex among women.

## Conclusion

According to the findings of the present study, associations of migraine with sex life issues were different for men and women. In this respect our findings are new and unique. The associations may be small or perhaps even clinically insignificant but they may make a great difference when improvements are applied in the lives they touch. GP has a vital role in the comprehensive treatment of migraine patients. Sex life is an essential part of health and investing in it will increase the overall well-being. In order to diminish suspicions and false beliefs GPs should ask about the sex life issues of their patients and offer related information to them when migraines are being treated.

## Competing interests

The author(s) declare that they have no competing interests.

## Authors' contributions

MPTS drafted the manuscript; AO and AR participated in drafting of the manuscript; LHS participated in the statistical analyses; KJM conceived of the study, and participated in its design and co-ordination. All authors have read and approved the final manuscript.

## Pre-publication history

The pre-publication history for this paper can be accessed here:


